# Use of simulation in teaching haematological aspects to undergraduate medical students improves student’s knowledge related to the taught theoretical underpinnings

**DOI:** 10.1186/s12909-021-02709-5

**Published:** 2021-05-12

**Authors:** Laila Alsuwaidi, Jorgen Kristensen, Amar HK, Saba Al Heialy

**Affiliations:** 1 College of Medicine, Mohammed Bin Rashid University of Medicine and Health Sciences, Dubai, United Arab Emirates; 2 Mediclinic City Hospital, Dubai Healthcare City Dubai, UAE; 3 Hamdan Bin Mohammed College of Dental Medicine, Mohammed Bin Rashid University of Medicine and Health Sciences, Dubai, UAE; 4 Meakins-Christie Laboratories, Research Institute of the McGill University Health Center, Montreal, Quebec Canada

**Keywords:** Simulation, Education, Medicine, Haematology, Immunology

## Abstract

**Background:**

Simulation is an educational method which has several modalities and applications. In the last few decades Simulation-Based Medical Education (SBME) has become a significant influence in medical education. Despite the recognized potential of simulation to be used widely in support of healthcare education, there are no studies focused on the role of simulation in teaching haematology. Moreover, the reaction level is the most commonly reported in medical education. This study evaluates, at two levels of Kirkpatrick’s model, the effectiveness of incorporating SBME in teaching haematological aspects to medical students.

**Methods:**

A total of 84 second year medical students from two cohorts received theoretical components of Haematopoietic and Immune System in 4 credits course, delivered using lecture approach. First cohort students (*n* = 49) participated in interactive learning tutorials to discuss clinical vignettes. Second cohort (*n* = 35) students participated in simulation sessions where the tutorial’s clinical vignettes were developed to clinical simulation scenarios conducted in the simulation centre. The potential influence of the simulation in learning enhancement was evaluated using Kirkpatrick’s Evaluation Framework.

**Results:**

The students rated the simulation sessions highly and found them to be a valuable learning experience. The category performance summary, generated by the assessment platform, demonstrates improvement in the student’s knowledge enhanced by the SBME.

**Conclusions:**

Adaptation of SBME in teaching haematological aspects is a feasible way to improve the student’s knowledge related to the taught theoretical foundations. SBME has the potential to enhance the undergraduate medical curriculum and it is expected, in the near future, to be an increasingly recommended educational strategy to bridge the gap between theory and practice.

## Background

Simulation is an interactive educational tool that is increasingly being incorporated into undergraduate medical education [1]. One of the distinct movements that have encouraged the development of clinical simulation is the medical education reform that has been driven by worldwide recognition of the need for students to be prepared as effective junior doctors after their undergraduate education [2, 3]. Moreover, the recognition of information overload within the undergraduate curriculum, at the expense of the learning of clinical and communication skills, encouraged the widespread adaptation of programs in clinical skills learning and the development of the educational facilities to support learning [4, 5].

There is growing literature reporting that simulation training is superior to traditional educational methods for training specific procedural skills like surgical procedures [6]. Studies show that simulation is beneficial for learners to improve behaviors and product skill outcomes in medical undergraduates as it provides learners with the opportunity to experience realistic clinical scenarios [7, 8]. Studies also demonstrated improved performance by undergraduate medical students in Objective Structured Clinical Examination (OSCE) assessments following simulation training [9]. Moreover, medical students consistently report that they find simulation training to be educationally beneficial, and it improves their confidence in challenging and uncertain situations [10, 11].

Evidence from SBME and health services research programs that are thematic, sustained, and cumulative shows that measured outcomes can be achieved at different levels including educational laboratory (T1), patient care practices (T2) and patient and public health (T3) [12].

The literature on simulation is growing rapidly, however, quantitative and qualitative research programs are needed to show how and why the results are achieved in different settings [13]. Increased scientific rigor is needed as well as improved data reporting conventions (e.g. report descriptive statistics and results of statistical tests), so that research outcomes can be replicated, synthesized quantitatively, and added to cumulative educational science [14]. Best Evidence Medical Education (BEME) review of effective learning through high-fidelity simulation identified only 109 articles (from 670) that were sufficiently robust to be included in the process [15]. The BEME review revealed that much of what has been and is being written in medical education literature is limited in scope to reporting evaluations, usually at the lower end of the Kirkpatrick criteria [16, 17].

**Fig. 1:**
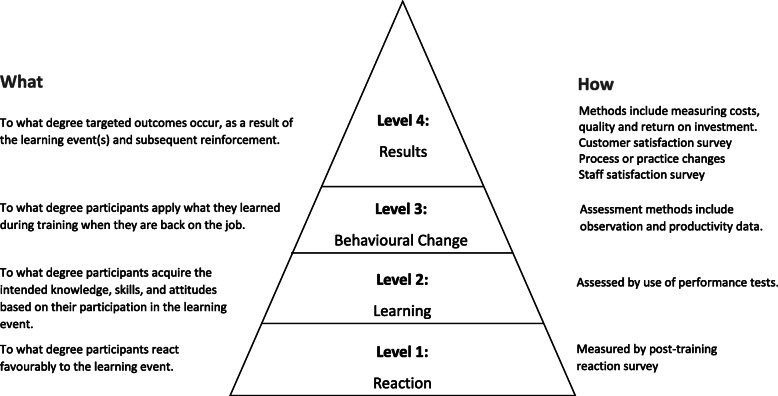
The Kirkpatrick four-level evaluation model

Kirkpatrick model is the most widely used framework for training program evaluation (Fig. 1). The model consists of four levels (reaction, learning, behavior, and results) and was developed in 1959 with several revisions made since. The model is designed to objectively measure the effectiveness of a training program, and strengths of Kirkpatrick’s model lie in its simplicity and pragmatic way of helping practitioners think about the programs and evaluate its impact [18]. The reaction level of the model presents the lower end of the Kirkpatrick criteria that is used to measure how engaged the participants were, how actively they contributed, and how they reacted to the training which helps to understand how well they received the training.

Despite the recognised potential of simulation to be used widely in support of health care education at all levels and across all disciplines, the reaction level is the most commonly reported in medical education. At the present, there are limited studies showing evidence that simulation training leads to better learning and knowledge improvement [16]. In fact simulation is seldom discussed within the education of basic sciences aspects to undergraduate medical students [19]. Most studies on the use of simulation in pre-clinical basic science education have focused on anatomy, physiology and pharmacology [20, 21]. A recent study on the use of simulation in immunology highlighted the role of simulation in enhancing OSCE scores post-simulation compared to pre-simulation in a cohort of 102 students [20]. To our knowledge, there are no studies focused on the role of simulation in the context of haematology teaching. We recognize this underutilization of simulation in haematology as an opportunity to integrate basic concepts with the practical application.

This study evaluates the effectiveness of incorporating simulation-based training, in teaching haematological aspects to second year medical students, in improving student’s knowledge related to the taught theoretical foundations and self-reported confidence. The present study quantifies students’ overall knowledge to determine whether undergraduate medical students participating in scenario-based simulation acquired more knowledge than case-based tutorials.

## Methods

### Study design

The study included second year medical students from two cohorts at Mohammed Bin Rashid University of Medicine and Health Sciences (MBRU) and took place at Khalaf Al Habtoor Medical Simulation Center in Dubai. The sample was homogenous with respect to age and the academic background standardized during the admission selection procedure. The two cohorts received the same sequence of the items in the syllabi and learning topic and taught by the same instructors who participated in all modalities and were involved in the design and implementation of the study. First cohort (*n* = 49) and second cohort (*n* = 35), from separate admission years, received theoretical component of Haematopoietic and Immune System in a four credits course, delivered using a mixture of approaches including lectures and flipped classroom. First cohort students participated in 9 case-based interactive tutorials to discuss clinical vignettes, each with two scenarios. Second cohort students participated in 6 case-based interactive tutorials and 3 scenario-based simulation sessions, each with 2 scenarios, where the tutorials’ clinical vignettes were developed to clinical scenarios conducted in the simulation center.

In each cohort, the students were divided into two sections to ensure that each section received enough time to complete the session. First cohort was divided into 8 groups: 7 groups of 6 students and one group of 7 students. The second cohort students attended the simulation centre three times within the course and were exposed to a variety of scenarios that addressed different haematological aspects including different types of anaemia, common bleeding disorders, and discuss the possible interventions for the case management and were required to interact with standardized patients (SPs). This cohort was divided into a total of 6 groups: 5 groups of 6 students and one group of 5 students. The instructors participated in all modalities of the study and are involved in the design and implementation of the study. Each group of students was exposed to 6 scenarios. One student per group volunteered to take part in each scenario. The active student was given a student information sheet of the scenario and given time to read before being introduced to the SP. The rest of the student’s group observed the scenario and interaction via a video capture. The students who were observing were required to critique the student participating in terms of their communication skills, the participant’s ability to address the issue in this case and whether they were in agreement with the outcome. The scenarios took place in a range of settings within the simulation centre. These included consulting rooms, wards and the ICU environment. Types of simulation settings and choice of learning objectives are described in Table 1. Each section was given an overall introduction to the day’s simulation activities and then the groups were allocated to their appropriate debrief room for a 5-minute pre-brief, followed by a 10-minute scenario and a 15-minute debrief before they moved onto their next scenario. Within the debrief component the students are guided by the facilitators to reflect on the scenario and decisions made using the theoretical knowledge gained in the earlier part of the semester. The Gibbs model of reflection is used to structure the de-briefs of all the scenarios. SPs were used in all 6 scenarios to act out a variety of roles. All SPs had previously received training and been assessed for their skill in playing a variety of roles. All SPs were included in the de-brief and invited to constructively contribute in terms of their perspective into what had gone well and what could have been improved. Scenarios were written by the content expert in line with the learning outcomes described in the course’s study guide. The simulation team reviewed and endorsed the final version.

**Table 1:** Summary of scenarios conducted in Haematopoietic & Immune System course

**Scenario number**	**Scenario Theme**	**Location Setting**	**Learning Objectives**
Haematology scenario 1	Vitamin B12 deficiency	Outpatient clinic	• Identify blood cells morphology in megaloblastic anaemia using peripheral blood films. • Correlate red blood cells morphology with other clinical features and laboratory findings. • Discuss the differential diagnosis of macrocytic anaemia. • Develop effective communication skills. Develop patient’s history taking skills.
Haematology scenario 2	Secondary anaemia	Outpatient clinic	• Diagnostic approach to a patient with anaemia • Classification of different types of anaemia • Discuss management of secondary anaemia in general terms • Improve the communication skills & establish a trust in a patient-physician relation. • Ensure that the patients receive full information in an understandable way about the diagnosis and plan.
Haematology scenario 3	Thrombocytopenia	Outpatient clinic	• Diagnostic approach to a patient with thrombocytopenia and that Immune Thrombocytopenia (ITP) is a diagnosis of exclusion. • Classification of different causes of thrombocytopenia • Discuss management of Immune Thrombocytopenia (ITP) in general terms • Improve the communication skills & establish a trust in a patient-physician relation. • Ensure that the patients receive full information in an understandable way about the diagnostic workup, diagnosis and plan.
Haematology scenario 4	Thrombophilia	Emergency	• Evaluate short case studies using the medical history and laboratory results to determine the likely diagnoses. • Interpret clinical and laboratory data as it relates to common bleeding disorders. • Discuss the possible interventions for the case management. • Develop effective communication skills & develop patient’s history taking skills.
Haematology scenario 5	Haemophilia	Delivery suite	• Evaluate short case studies using the medical history and laboratory results to determine the likely diagnoses. • Interpret clinical and laboratory data as it relates to common bleeding disorders. • Discuss the possible interventions for the case management. • Develop effective communication skills & develop patient’s history taking skills.
Haematology scenario 6	Sideroblastic in progress	Outpatient clinic	• Diagnostic approach to a patient with anaemia. • Discuss the differential diagnosis of anaemia. • Discuss management of anaemia in general terms • Improve the communication skills & establish a trust in a patient-physician relation. • Improve patient’s history taking skills.

### Measurement

The outcome measures related to the potential influence of the simulation in learning enhancement was evaluated at two levels of Kirkpatrick’s Evaluation Framework: (1) reaction level using student post-session satisfaction feedback questioner, (2) learning level through knowledge test using the analysis feature of the exam platform ‘Examsoft’ to demonstrate whether the simulation has impact on the students’ knowledge.

#### Reaction level

At the end of every session, students were asked to complete a pilot satisfaction feedback form. Participants were asked to select the relevant words that best describe the session and to what degree they were satisfied with key elements of the simulation training. Students had the opportunity within the questionnaire to choose words from a word cloud format which contained an equal number of positive and negative words. The positioning of the words changed to ensure that there was no bias to positive statements. The questionnaire is administrated to measure the perceptions, reactions and attitude of the students about the effectiveness of the training including logistic support.

#### Learning level

The course has an inbuilt formal evaluation guided by blueprint for the learning topics (Table 2). The evaluation measures the students’ knowledge gain and the achievement of the ultimate course objectives. During pre-simulation evaluation, a comprehensive, quiz-type knowledge test is administered to assess the initial level of the students’ knowledge. This test is prepared with more knowledge-type and some understanding and skill-oriented questions. At the end of the course, post-simulation test is administered to measure the student’s knowledge gain.

**Table 2:** Aspects tested in pre and post-learning knowledge assessments

**S. No.**	**Category**
1	Anatomy of blood and function of its major components
2	Anaemia: causes, classification, clinical features and diagnosis
3	Erythropoiesis: definition, stages and mature red blood cells features
4	General aspects of red blood cell: haemoglobin, methemoglobin and cell membrane
5	Genetic disorders of haemoglobin
6	Haematopoiesis: definition, sites and general aspects of blood formation and maturation
7	Hypochromic anaemias: features, cause and differential diagnosis
8	Iron deficiency anaemia: clinical features, causes, diagnosis and management
9	Nutritional and metabolic aspects of iron
10	Thalassaemia: definition, types, geographical distribution, diagnosis and management

### Statistical Analysis

Using the exam platform ‘Examsoft’ the category performance analysis report is generated to complete the quantitative data analysis. Data was entered in computer using IBM-SPSS for windows version 25.0 (SPSS Inc., Chicago, IL). The paired *t*-test was applied after testing the normality of data using Shapiro-Wilk test. The numbers of items by topic were used to adjust the percent of the correct answer before the test. *P*-value of 0.05 was use as a level of significant in all tests.

## Results

### Satisfaction feedback

**Fig. 2:**
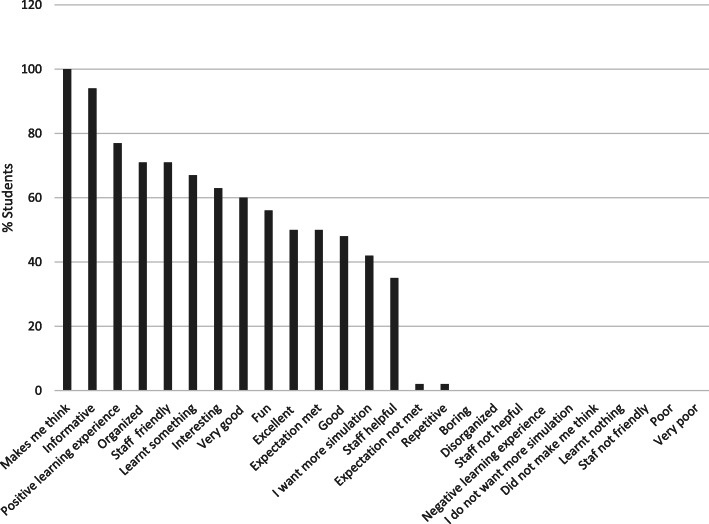
Students feedback on the post-training reaction survey

The results from the post-session satisfaction feedback questioner showed that 100 % of the students stated the simulation scenarios made them think and helped to support the theory already learnt in class, 94 % reported that the sessions were informative and 77 % found them a positive learning experience. More than 60 % of the students agreed that the simulation scenarios were interesting and added to their knowledge and no negative words were highlighted. (Fig. 2)

### Pre and post-course performance tests

**Fig. 3:**
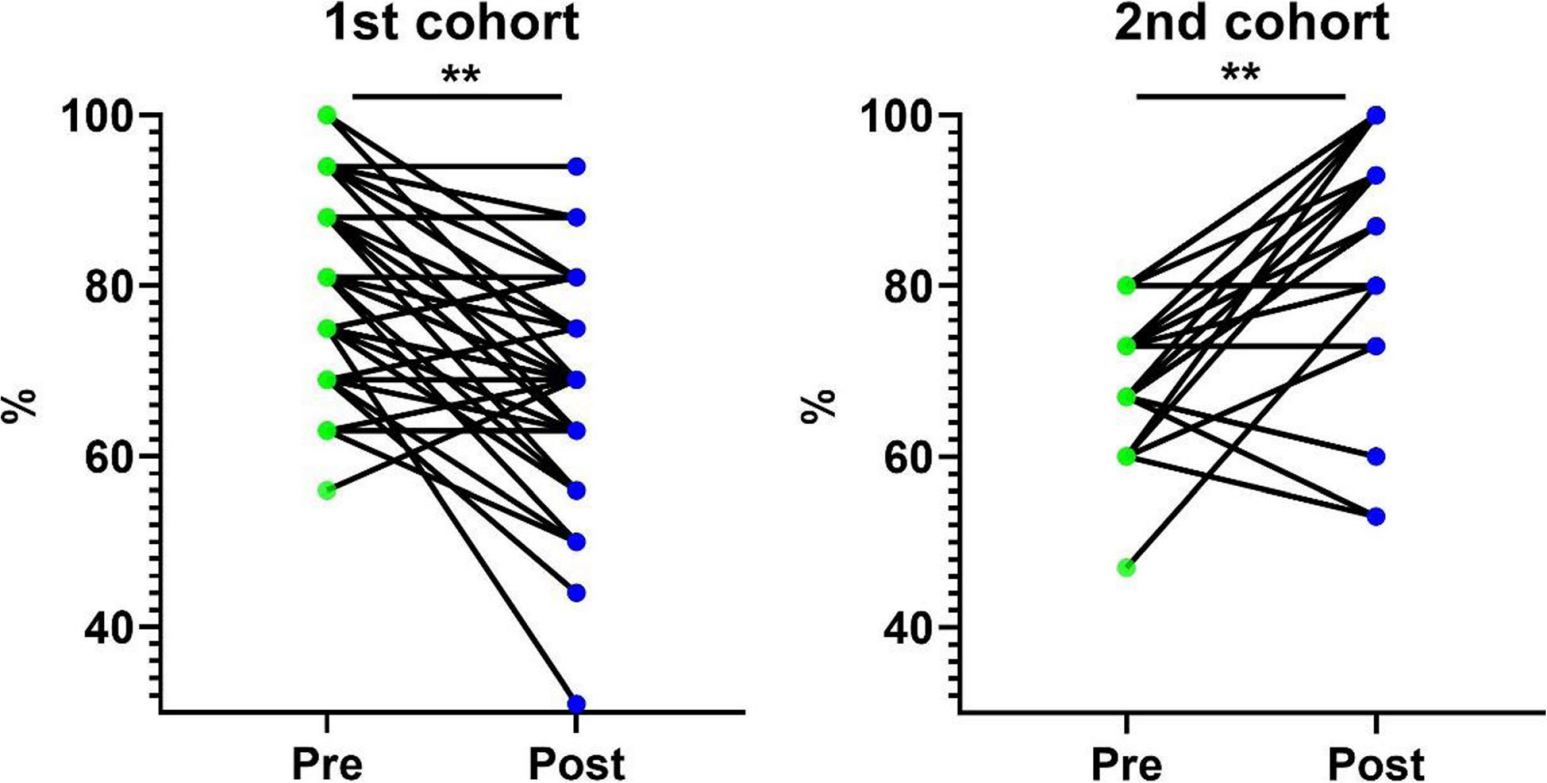
Comparison between pre- and post-tests in 1st and 2nd cohorts

The study was carried out on additional level of Kirkpatrick model. The second level of evaluation involves performance testing by determining the extent to which learning has occurred. The sample was homogenous with respect to age and the academic background standardized during the admission selection procedure. The two cohorts received the same syllabi and same sequence of learning topic and taught by the same instructors. The paired *t*-test was applied after testing the normality of data using Shapiro-Wilk test. The results for pre and post tests were assessed by examining the questions related to the 10 categories mentioned in Table 2 and were calculated as a percentage. The results in Table 3 show that the mean pre and post-course tests for the first cohort, the group without simulation, were 80.88 (± 1.50) and 68.12 (± 1.74) respectively. This was statistically significant (*p* = 0.001). The rate of change among the group from the post-test was − 15.11 %. Also, the analyses show that the mean of pre and post-tests for the second cohort, the group with simulation, were 69.94 (± 1.30) and 85.86 (± 2.32) respectively. There was a statistically significant change between the pre and post-tests (*p* = 0.006). Figure 3 shows the distribution of the students in the first cohort (*n* = 49) and the second cohort (*n* = 35) for the pre and post-test results. There was a statistically significant change between the pre and post-tests in both cohorts where the second cohort, the group with simulation, showed a significant increase in the post-test score. The rate of change among this group was 23.46 % (Table 4).

**Table 3:** Descriptive Statistical analysis

Paired sample statistics (*t*-test)		Mean	N	Std. Deviation	Std. Error Mean	Correlation	Sig.
1^st^ cohort (without simulation)	Pre-test	80.88	10	10.47	1.50	0.4264	0.001
	Post-test	68.12	10	12.17	1.74		
2^nd^ cohort (with simulation)	Pre-test	69.94	10	7.70	1.30	0.4164	0.006
	Post-test	85.86	10	13.75	2.32

**Table 4:** Comparison of the rate of change

		Mean	Std. Deviation	Std. Error Mean	
					Sig. (2-tailed)
1^st^ cohort (without simulation)	Pre-test & Post-test	-15.11	15.33	2.19	<0.001
2^nd^ cohort (with simulation)	Pre-test & Post-test	23.46	20.51	3.46	

The statistical analyses of category performance summary demonstrated that the rate of change among the group with simulation (23.46 %) was significantly higher than the rate of change among the group without simulation (-15.11 %) and the paired sample test for the differences was significant (*p* < 0.001). These results revealed that the simulation makes significant higher rate of change in the post-test result.

## Discussion

The evaluation of any training system helps to determine the value and effectiveness of a learning program. The evaluation can be measured using what is called the ‘knowledge gap’, the gap between what the trainer teaches and what the trainee learns as defined by Riech [21]. The most influential framework for the evaluation of training programs has come from Kirkpatrick’s model that follows a goal-based approach and built on four simple questions that translate into four levels of evaluation [18, 22].

In this study, we used two levels of Kirkpatrick’s model to quantify students’ overall knowledge to determine whether undergraduate medical students participating in scenario-based simulation acquired more knowledge, more than case-based tutorials. The trainees are undergraduate MBBS students and the objective of the course is to impart knowledge on concepts and skills related to diagnostic approaches in haematology and Immunology.

At level one of Kirkpatrick evaluation, data on the reactions of the participants at the end of the training program were gathered. This level is often measured with attitude questionnaires that are passed out after most training classes. This level measures the learner’s perception (reaction) of the course. Level one of Kirkpatrick evaluation assists in assessing participant’s reactions to a course’s instructor, setting, materials, and learning activities. The strength of this level of training evaluation is the ease of obtaining the information and it involves gaining direct feedback. However, positive satisfaction numbers do not ensure learning and subsequent application of program content.

In the current study, students rated the simulation sessions highly and found it a valuable learning experience. From an instructor’s point of view, it is important to get good satisfaction ratings. If participants are not satisfied, they probably will not be motivated to learn. So, while good satisfaction ratings do not guarantee learning, bad ratings most likely decrease the probability of it occurring [23].

The intention at level 2 of Kirkpatrick’s model is to assess whether the learning objectives for the program are met, have the students display actual knowledge of the subject before and after instruction. This is usually done by means of an appropriate test or examination. The learning evaluation requires post-testing to ascertain what knowledge was learned during the training, bearing in mind that the post-testing is only valid when combined with pre-testing. This is to differentiate between what students already knew prior to the training and what they learned during the training program.

Based on the above argument it has been obviously understood that the evaluation should go beyond immediate reactions of the attendees, therefore, the study was carried out on additional level of Kirkpatrick model. In the second level of evaluation, the statistical analyses of category performance summary of the assessment platform confirmed that the learning objectives were achieved in the cohort which received the simulation. Compared to their performance in the initial in-course assessment (pre-test), the performance in the final assessment (post-test) decreased (*p* = 0.001) for the cohort without simulation and the cohort with simulation increased (*p* = 0.006). Moreover, the rate of change among the group with simulation was significantly higher than the rate of change among the group without simulation and the paired sample test for the differences was significant (*p* < 0.001), which demonstrates improvement in the student’s knowledge enhanced by the simulation. It would seem that with the cohort which did not receive the simulation the retention of the information was not as effective. This would be an important question to address in the future.

A limitation of this study could be the use of Gibbs model for de-briefing. This model was chosen by the institution as it is one of the most established models of reflection. It is easy to use and understand. It is an open structure and can be applied to many disciplines. However, a disadvantage of the Gibbs model of reflection is that it may be descriptive [24]. Therefore, it may be worthwhile exploring other models of reflection in the future.

## Conclusions

The present study reports for the first time the effectiveness of incorporating simulation-based training in teaching haematological aspects to undergraduate medical students to improve student’s knowledge related to the taught theoretical underpinnings and to improve self-perceived competency. The study shows that undergraduate medical students participating in scenario-based simulation acquired more knowledge than case-based tutorials. Adaptation of SBME in teaching haematological aspects to second year medical students is a feasible way to improve the student’s knowledge related to the taught theoretical foundations. SBME has the potential to enhance the undergraduate medical curriculum and it is expected in the near future to be an increasingly recommended educational strategy to bridge the gap between theory and practice.

However, it should be noted that although a participant may possess the knowledge, skills, and attitudes taught in the course, there is still no guarantee of on-the-job application of acquired knowledge and skills. There is a need for robust research that are focused on higher level outcomes in order to provide convincing evidence across the whole spectrum of the efficacy and effectiveness of simulation-based education.

## Data Availability

The datasets generated and/or analyzed during the current study are not publicly available as they form a part of the student assessment record and feedback for individual course at MBRU but are available from the corresponding author on reasonable request.

## Abbreviations

SBME: Simulation-based medical education
OSCE: Objective structured clinical examination
BEME: Best evidence medical education
MBRU: Mohammed Bin Rashid University of medicine and health sciences
SPs: Standardized patients
